# Cold Exposure Alleviates Colitis via Parallel Integration of Colonic Mucosal Regeneration and Ileal Antimicrobial Defense

**DOI:** 10.3390/biomedicines14030609

**Published:** 2026-03-09

**Authors:** Yuzhu Di, Jiaxing Deng, Ziyou Hong, Zhirui Liu, Lubo Jin, Wenyuan Zhao, Bo Qu

**Affiliations:** 1 Department of Gastroenterology and Hepatology, The Second Affiliated Hospital of Harbin Medical University, Harbin 150000, China; yuzhudi@hrbmu.edu.cn (Y.D.);; 2 College of Bioinformatics Science and Technology, Harbin Medical University, Harbin 150086, China; djx245056106@163.com (J.D.);

**Keywords:** cold exposure, inflammatory bowel disease, colonic stem cells, antimicrobial peptides

## Abstract

**Background:** Inflammatory bowel disease (IBD) involves chronic intestinal inflammation, epithelial barrier disruption, and dysbiosis, with environmental factors playing a significant role in its pathogenesis. Previous work revealed that cold exposure alleviates colitis in mice; this study extends that finding by demonstrating that cold exposure enhances intestinal regeneration even in healthy mice, upregulating proliferation markers (Mki67, PCNA, Cyclin D1). **Methods:** Applying this pro-regenerative effect to a colitis model, we investigated the underlying mechanisms through multi-omics analysis, transmission electron microscopy (TEM), immunofluorescence, and pathological staining as well as 16S rRNA sequencing. **Results:** We found that cold exposure activates intestinal epithelial proliferation pathways. Further analysis indicated that cold exposure induces colonic stem cell regeneration, upregulating stem cell markers Lgr5 and Ascl2. Notably, colonic transcriptomic profiling revealed the emergence of a Paneth-like cell phenotype, characterized by altered expression of specific lineage genes. Furthermore, cold exposure simultaneously promoted the accumulation of secretory granules and upregulated the expression of antimicrobial peptide genes (such as Lysozyme and Defa) in ileal Paneth cells. This enhanced ileal antimicrobial defense effectively reshaped the gut microbiota in inflamed intestines. **Conclusions:** This research elucidates a mechanism whereby cold adaptation promotes mucosal repair by integrating localized colonic epithelial regeneration with enhanced ileal Paneth cell-mediated antimicrobial defense. This offers compelling new perspectives on how environmental factors, such as cold exposure, could influence the pathophysiology of IBD and contribute to intestinal regeneration, which may provide foundational theoretical support for the future diagnosis and treatment of IBD.

## 1. Introduction

Inflammatory bowel disease (IBD) is a group of chronic, relapsing inflammatory disorders of the gastrointestinal tract, primarily comprising ulcerative colitis (UC) and Crohn’s disease (CD) [1,2]. Characterized by prolonged and recurrent intestinal inflammation, IBD poses a significant global health burden. According to the Global Burden of Disease (GBD) 2021 study, approximately 3.83 million people were living with IBD worldwide, and the disease was responsible for about 1.51 million disability-adjusted life years (DALYs) [3]. In recent decades, the epidemiological pattern of IBD has evolved in parallel with industrialization and ecological modernization. Notably, developing countries and newly industrialized regions across Asia and Africa have observed a rising incidence, mirroring earlier trends in Western nations. From the 1990s to the early 21st century, the global incidence of IBD continued to increase [4], accompanied by a growing risk of colorectal cancer.

A hallmark of IBD is the disruption of gut microbiota homeostasis in genetically susceptible individuals, triggered by environmental factors. This dysbiosis is marked by an increase in pathogenic bacteria and alterations in the differentiation or exhaustion of intestinal stem cells. Persistent mucosal chronic inflammation leads to the overactivation of immune responses, which in turn exacerbates epithelial damage [5]. Current management strategies often involve immunosuppressive agents [6]. However, the clinical utility of these treatments is constrained by relatively high rates of non-response and severe side effects [7]. Therefore, elucidating the pathogenic mechanisms of IBD and developing effective therapeutic interventions are of great importance for disease prevention and treatment.

The intestine exists as a dynamic environment open to the external world and is constantly exposed to a variety of physiological, pathological, and environmental stimuli [8]. When the intensity of these stimuli exceeds the intestine’s capacity to maintain homeostasis, IBD may be triggered. Current research indicates that the pathogenesis of IBD is closely associated with genetic and environmental factors, and gene–environment interactions (G × E) also play an important role in the onset and progression of the disease [9]. Common mechanisms through which G × E influences IBD include environmental exposures, genetic susceptibility, immune dysregulation, and disturbances in the gut ecosystem [10]. Among these, environmental factors—increasingly influenced by modern industrialization—have attracted considerable attention in recent years. A study by Neamţi et al. reported that disease activity in IBD patients often peaks in spring and autumn, with most inflammatory markers showing lower levels in winter [11], suggesting a potential link between IBD and climatic conditions. Temperature, as a key climatic variable, has so far received limited attention in IBD research. Studies on other diseases have revealed important roles for temperature variation. For example, Jurado-Fasoli et al. demonstrated that cold exposure alters signaling lipid levels in young adults, yielding beneficial effects on cardiometabolic health [12]. Similarly, Takahiro Seki et al. provided evidence that low temperatures markedly improve metabolic disorders in mice and suppress colorectal tumor development by diverting energy toward thermogenesis [13]. In the context of inflammatory diseases, Conti et al. found that cold exposure inhibits NF-κB signaling and reduces circulating IL-6, ameliorating insulin resistance in prediabetic models and indicating a protective effect against inflammatory and metabolic disorders [14]. Martina Spiljar et al. showed that cold treatment shifts the energy balance between autoimmunity and thermogenesis in mice, reduces MHCII expression on monocytes, and attenuates neuroinflammation by limiting autoreactive T-cell activation [15].

Building on our previous work, we were the first to identify that cold exposure, as an independent protective factor, significantly attenuates chemically-induced colonic inflammation in mice. The study established a novel link between cold intervention and biological changes in colonic inflammation, yet the precise regulatory mechanisms remained unclear. Our current investigation expands upon previous research by integrating multi-omics technologies with comprehensive molecular validation. We demonstrate that systemic cold exposure functions as a physiological primer that mitigates colonic inflammation through a synergistic multi-organ strategy: on the one hand, it locally enhances the expansion of colonic stem cells to accelerate mucosal healing, accompanied by alterations in the gene expression profile toward a “Paneth-like” secretory phenotype. On the other hand, it induces an increase in Paneth cell secretory granules within the ileum, which leads to upregulated expression of antimicrobial peptides and the subsequent remodeling of the gut microbiota. By elucidating this cold-induced systemic fortification mechanism of the intestinal barrier, our findings provide a novel theoretical basis for developing region-specific IBD healthcare strategies and offer insights into preventive interventions against chronic inflammatory diseases in cold regions.

## 2. Materials and Methods

### 2.1. Establishment of the Mouse Cold Stress Model

Male C57BL/6J mice were obtained from the Experimental Animal Center of the Second Affiliated Hospital of Harbin Medical University (Ethical Approval No: SYDW2023067). A mouse model of cold exposure was established as previously described [16]. Briefly, 10-week-old mice (*n* = 10 per group) were housed under a 12-h light/12-h dark cycle with free access to food and water at a controlled ambient temperature of 22 ± 3 °C. The mice were randomly divided into a control group and a cold-exposed group. The control group was continuously maintained at 22 ± 3 °C. Mice in the cold-exposed group were subjected to a moderate cold environment (4 ± 1 °C) in a climatic chamber (Tianling Co., Cangzhou, China) for 8 h per day during nighttime (dark cycle). This regimen was continued for three consecutive weeks (21 days). This specific mouse model was established based on the authoritative cold exposure protocol reported in the recent Nature [13] and our own preliminary condition-optimization experiments [16].

### 2.2. Mouse Models of Colitis

For the colitis models, male C57BL/6J mice (*n* = 8 per group) were used. Colitis was induced based on a previously reported method with minor modifications [17]. To align the total duration of cold exposure with the healthy cold stress model (21 days), the cold-exposed colitis group (DSS + CE) underwent a 26-day protocol involving an initial 14-day cold preconditioning phase (4 ± 1 °C for 8 h nightly), followed by 7 days of 2% dextran sulfate sodium (DSS) administration combined with continued cold exposure, then 3 additional days of DSS administration at normal room temperature, and finally, a 2-day recovery period with regular drinking water. The colitis-only control group (DSS) received the same 10-day DSS treatment and 2-day recovery but was maintained at normal room temperature throughout. Body weight and disease activity index (DAI) were monitored daily [18]. Upon euthanasia, colon tissues and serum samples were collected for subsequent analysis.

### 2.3. Isolation of Intestinal Epithelial Cells

The primary intestinal epithelial cells were isolated as described previously [19]. Briefly, the collected colon was cleared of adherent adipose tissue, rinsed with PBS, and opened longitudinally. The tissue was then cut into approximately 0.5 cm pieces after incubation in pre-cooled PBS containing 2% FBS with vigorous shaking. The tissue fragments were incubated in 10 mL of pre-digestion solution at 37 °C for 15 min with gentle agitation. The suspension was subsequently vortexed vigorously, and the supernatant was collected. Steps 3 and 4 were repeated to maximize yield. The combined supernatant was filtered to remove large tissue debris and centrifuged at 1500 rpm for 5 min at 4 °C. The resulting pellet was resuspended in an appropriate buffer for subsequent analysis. The digestion solution consisted of 50 mL of 5 mM EDTA, 18.5 mL of 54 mM HEPES, and 31.5 mL of HBSS.

### 2.4. RNA Sequencing Data Analysis

Total RNA was extracted from the isolated primary ileal epithelial cells) and colonic epithelial cells using the Trizol reagent. The RNA concentration and purity were assessed using a NanoDrop 2000 spectrophotometer (Thermo Fisher Scientific, Wilmington, DE, USA). Transcriptome sequencing was performed with the assistance of OE Biotech Co., Ltd. (Shanghai, China). Briefly, mRNA-Seq libraries were constructed from qualified total RNA samples using the Illumina TruSeq RNA Sample Preparation Kit (Illumina, Inc., San Diego, CA, USA). These libraries were then sequenced on an Illumina HiSeq 2500 platform to generate paired-end reads with a length of 50 bp (2 × 50 bp).

### 2.5. Differential Expression Analysis

Differential expression analysis was performed using the “limma” package [20]. Raw RNA-Seq counts were normalized and variance-stabilized using the voom method, which transforms data into log2-counts per million (log2-CPM) for linear modeling. Linear models were then fitted with an empirical Bayes approach to estimate log2-fold changes (log2FC) and their statistical significance. *p*-Values were adjusted using the Benjamini–Hochberg method [21], and differentially expressed genes were defined by an FDR < 0.05 and |log2FC| > 1.

### 2.6. 16S rRNA Gene Sequencing and Microbiota Analysis

Total genomic DNA was extracted from the colonic contents using a QIAamp DNA Stool Mini Kit (Qiagen, Hilden, Germany). The DNA concentration and integrity were assessed by a NanoDrop 2000 spectrophotometer and 1% agarose gel electrophoresis. 16S rRNA gene sequencing was performed with the assistance of OE Biotech Co., Ltd. (Shanghai, China). Briefly, the V3–V4 hypervariable regions of the bacterial 16S rRNA gene were targeted for PCR amplification. The resulting amplicons were purified, quantified, and used to construct sequencing libraries using the Illumina TruSeq DNA Sample Preparation Kit. These libraries were then sequenced on an Illumina NovaSeq platform to generate 250 bp paired-end reads. Raw sequencing data were processed and analyzed using the QIIME2 software package, including taxonomic assignment against the SILVA database.

### 2.7. Hierarchical Clustering

To visualize the expression patterns of selected genes across sample groups, Z-score normalization was applied. The resulting values were used for hierarchical clustering based on Euclidean distance with the “pheatmap” function in R. The cluster result was displayed as a heatmap, where a consistent color scale represents relative gene expression levels.

### 2.8. Functional Enrichment Analysis

KEGG (Kyoto Encyclopedia of Genes and Genomes) pathway enrichment analysis of the differentially expressed genes was conducted using the “clusterProfiler” R package [22] to explore their potential biological functions. Pathways with a *p*-value < 0.05 were considered statistically significant.

### 2.9. Gene Set Enrichment Analysis (GSEA)

GSEA [23] was employed to determine whether predefined gene sets exhibited statistically significant differences between two biological states (e.g., colon vs. cold-exposed colon). Using gene sets from the Molecular Signatures Database (MSigDB) v7.4, enrichment scores (ESs) were calculated and normalized to generate normalized enrichment scores (NESs). Gene sets with |NES| > 1 and a nominal *p*-value < 0.05 were deemed statistically significant.

### 2.10. H&E Staining

Colonic tissues were fixed in 4% paraformaldehyde, then paraffin-sectioned and incubated at 60 °C for 2 h. Sections were gradient-deparaffinized, stained with hematoxylin for 30 min, rinsed with deionized water, treated with a differentiation solution for 30 s followed by eosin staining for 2 min, rinsed again with deionized water, and soaked for 5 min. After rapid gradient dehydration, sections were mounted with neutral resin, dried at 60 °C, and microscopically photographed.

### 2.11. Immunofluorescence Staining

Paraffin-embedded colon tissue sections were deparaffinized, rehydrated, and subjected to antigen retrieval using a microwave with sodium citrate buffer (pH 6.0). The sections were then blocked with an appropriate blocking solution for 1 h at room temperature. Subsequently, the sections were incubated overnight at 4 °C with the following primary antibodies: anti-EpCAM (21050-1-AP, Proteintech, Rosemont, IL, USA), anti-Mki67 (Proteintech), anti-PCNA (BM0104, Boster, Pleasanton, CA, USA), and anti-F4/80 (#30325, CST, Danvers, MA, USA). After washing with PBS, the sections were incubated with species-matched fluorescently-labeled secondary antibodies at room temperature for 2 h in the dark. Nuclei were counterstained with DAPI (C1002, Beyotime, Shanghai, China). High-resolution images were acquired using a high-resolution slide scanner (3DHISTECH Ltd., Budapest, Hungary), and fluorescence signal analysis was performed using ImageJ software (NIH, Bethesda, MD, USA; version 1.54s).

### 2.12. Transmission Electron Microscopy

To evaluate the ultrastructure of Paneth cells and their secretory granules, intestinal tissue samples were processed for transmission electron microscopy. Briefly, freshly dissected small intestinal segments were immediately fixed in 2.5% glutaraldehyde in 0.1 M phosphate buffer (pH 7.4) at 4 °C for at least 4 h. After washing, the samples were post-fixed in 1% osmium tetroxide, dehydrated through a graded ethanol series, and embedded in Spurr’s epoxy resin. Ultrathin sections (70–90 nm) were prepared using an ultramicrotome, mounted on copper grids, and double-stained with uranyl acetate and lead citrate. The specimens were ultimately observed and imaged under a Hitachi HT7700 transmission electron microscope (Tokyo, Japan) operated at 80 kV.

### 2.13. Cell Cycle Analysis by Flow Cytometry

The cell cycle distribution of mouse colonic epithelial cells was analyzed using a Cell Cycle Detection Kit (C1052, Beyotime) according to the manufacturer’s instructions, with minor adaptations. Briefly, harvested cells were fixed in 1 mL of ice-cold 70% ethanol at 4 °C overnight. After fixation, cells were centrifuged and the ethanol was thoroughly removed. The cell pellet was washed once with cold phosphate-buffered saline (PBS) and then resuspended in 0.5 mL of the staining solution provided in the kit, which contains propidium iodide (PI) and RNase A. The mixture was incubated in the dark at 37 °C for 30 min. Flow cytometric analysis was immediately performed using a BD FACSCantoII flow cytometer (San Jose, CA, USA). Data from a minimum of 20,000 single-cell events per sample were collected, and the cell cycle phases (G1, G2, and S) were quantified using ModFit LT analysis software (Verity Software House, Topsham, ME, USA; version 6.0).

### 2.14. Enzyme-Linked Immunosorbent Assay (ELISA)

The concentrations of inflammatory cytokines TNF-α and IL-1β in mouse serum were quantified using commercially available ELISA kits from Jianglai Biotechnology (Shanghai, China) according to the manufacturer’s instructions. Briefly, blood samples were collected immediately after euthanasia, and serum was separated by centrifugation. Standards, controls, and serum samples were added to the pre-coated plates and incubated at 37 °C for 90 min. After washing, the biotinylated detection antibody was added, followed by incubation with horseradish peroxidase-conjugated streptavidin. Following thorough washing, TMB substrate was added for color development in the dark, and the reaction was stopped with stop solution. The absorbance was measured at 450 nm using a microplate reader. Sample concentrations were determined based on the standard curve, and all samples were assayed in duplicate with at least three independent experiments.

### 2.15. Quantitative Real-Time PCR (qRT-PCR)

Total RNA was extracted using the TransZol Up Plus RNA Kit (TransGene, Beijing, China, ER501-01-V2), and its concentration and purity were assessed. Reverse transcription was carried out with a TIANGEN Kit (Beijing, China, KR118) to synthesize cDNA. Quantitative real-time PCR amplifications were performed in triplicate using SYBR Green mix (TransGene, AQ141-01), with 1 μL of cDNA per reaction. Gene expression levels were calculated by the 2^−ΔΔCT^ method, and results represent the mean of at least three independent biological replicates. The sequences of all primers used for qRT-PCR are listed in Supplementary Table S4.

### 2.16. Western Blotting

Total protein was extracted from primary intestinal epithelial cells subjected to various treatments for the indicated durations. Cells were lysed on ice for 30 min using RIPA buffer (50 mM Tris-HCl, pH 7.4, 150 mM NaCl, 1% Triton X-100, 2 mM EDTA, 0.1% SDS, 5 mM sodium orthovanadate) supplemented with 1x protease inhibitor cocktail (Roche Molecular Biochemicals, Basel, Switzerland). Protein concentration was quantified with a BCA Protein Assay Kit (Beyotime, China). Equal amounts of protein lysates were separated by SDS–PAGE and subsequently transferred onto nitrocellulose membranes (Cytiva, Marlborough, MA, USA). The membranes were blocked with 5% fish skin gelatin in TBST for 1 h at room temperature and then incubated with the following primary antibodies for 2 h: anti-Cyclin D1 (60186-1-lg, Proteintech), anti-PCNA (15014-1-AP, Proteintech), anti-Lgr5 (30007-1-AP, Proteintech), anti-Ascl2 (21368-1-AP, Proteintech), and anti-β-actin (TA-09, Zhongshan Goldenbridge-Bio, Beijing, China). After three washes with TBST, the membranes were probed with DyLight 800-conjugated goat anti-mouse or anti-rabbit secondary antibodies (Kirkegaard & Perry Laboratories, Gaithersburg, MD, USA) at a 1:10,000 dilution for 1 h. Protein bands were visualized and quantified using an Odyssey Infrared Imaging System and its accompanying Odyssey 2.1 software (LI-COR Biosciences, Lincoln, NE, USA).

### 2.17. Statistical Analysis

All bioinformatics analyses were performed using R software (version 4.4.2). All statistical tests were two-sided and adjusted for multiple comparisons using the Benjamini–Hochberg method for false discovery rate (FDR) control, with a significance threshold defined as FDR < 0.05. All in vivo experiments were performed with at least three biological replicates, each derived from independent biological samples. Data were analyzed using the Student’s *t*-test, one-way ANOVA, or two-way ANOVA followed by Tukey’s HSD post hoc test for multiple comparisons with GraphPad Prism software (GraphPad Software, Boston, MA, USA; version 11.0.0). For α-diversity indices (Observed, Chao1, ACE, Shannon, Simpson, Faith’s PD), the Kruskal–Wallis test was applied, followed by the Wilcoxon rank-sum test for pairwise comparisons. Beta diversity was assessed using principal coordinate analysis (PCoA) and non-metric multidimensional scaling (NMDS) based on Bray–Curtis distance, with group differences evaluated by PERMANOVA (adonis2 with 999 permutations). Detailed statistical information for each experiment is provided in the figure legends. A *p*-value of less than 0.05 was set as the threshold for statistical significance.

## 3. Results

### 3.1. Cold Exposure Upregulates Proliferative Signaling in Healthy Mouse Intestinal Epithelial Cells

**Figure 1:**
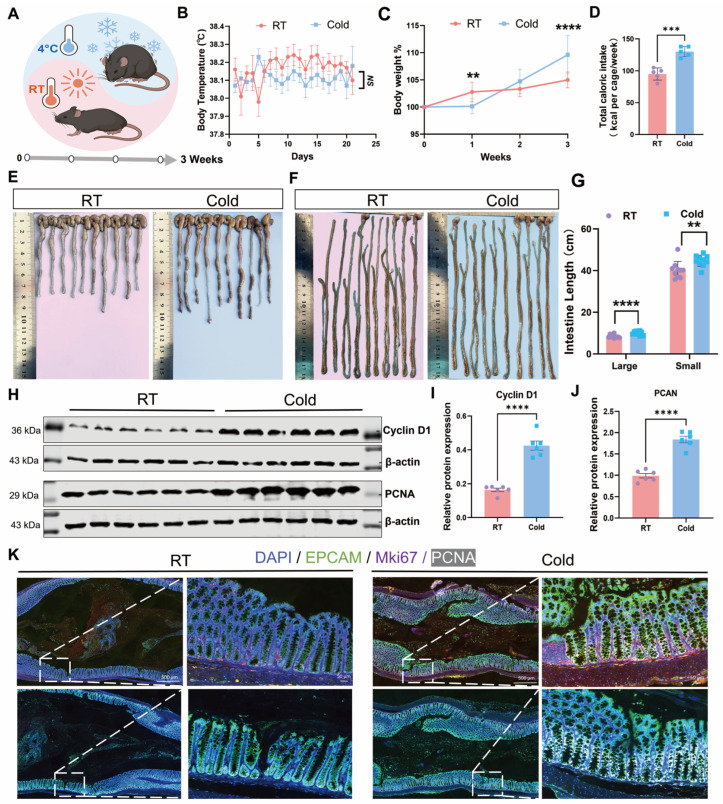
Cold exposure induces intestinal epithelial proliferation in mice. (**A**) Schematic diagram of the experimental timeline. Ten-week-old mice were subjected to cold exposure (4 °C) for 8 h per day over a period of three weeks, followed by tissue collection. (**B**) Daily rectal temperature measurements of mice in the room temperature (RT) and cold exposure (Cold) groups (*n* = 10). (**C**). Body weight changes of mice in the RT and Cold groups monitored weekly throughout the three-week period (*n* = 10). (**D**) Average caloric intake of mice cage in the RT and Cold groups, two mice in one cage (*n* = 5). (**E**,**F**) Representative images of the colon (**E**) and small intestine (**F**) from RT and Cold group mice. (**G**) Quantitative analysis of colon and small intestine lengths from (**E**,**F**) (*n* = 10). (**H**–**J**) Representative images of western blot analysis (**H**) and corresponding quantitative densitometry (**I**) of PCNA and (**J**) Cyclin D1 protein levels in isolated intestinal epithelial cells from different group mice (*n* = 6). (**K**) Representative immunofluorescence images of colon sections stained for the proliferation markers MKi67 (red) and PCNA (magenta), and the epithelial marker EPCAM (green). Nuclei are counterstained with DAPI (blue). All data represent means ± SEM. Statistical significance was determined by the unpaired two-tailed Student’s *t*-test ** *p* < 0.01, *** *p* < 0.001, **** *p* < 0.0001 *ns*, no significance.

Ten-week-old male mice were subjected to 4 °C cold exposure for 8 h daily over three weeks, followed by humane euthanasia and the collection of colon and small intestine tissues for subsequent analysis (Figure 1A). Core body temperature was monitored daily in both the room temperature (RT) and cold exposure (Cold) groups. Mice in the Cold group were measured after 30-minute acclimation at room temperature following removal from the cold chamber. As shown in Figure 1B, no statistically significant difference in body temperature was observed between the two groups. Body weight was tracked throughout the three-week period, revealing a significant decrease in the Cold group after one week of treatment, followed by a notable weight increase with prolonged exposure (Figure 1C). To investigate this metabolic adaptation, we measured caloric intake and found that cold-exposed mice consumed significantly more food per cage compared to the RT controls (Figure 1D). Morphometric analysis of intestinal tissues demonstrated a significant increase in both the colon and small intestine length in the Cold group versus the RT controls (Figure 1E–G). This intriguing finding suggests a cold-induced pro-proliferative phenotype in the intestinal epithelium. Subsequent Western blot analysis of the primary colonic epithelial cells revealed markedly elevated protein levels of the proliferation markers PCNA and Cyclin D1 in cold-exposed mice (Figure 1H–J), providing molecular evidence for enhanced proliferative signaling. Further validation via immunohistochemical staining of paraffin-embedded colon sections showed intensified EPCAM signal (green fluorescence) in the Cold group, confirming intestinal epithelial identity. Co-staining with proliferation markers Mki67 and PCNA (red and pink fluorescence, respectively) demonstrated enhanced signals that co-localized with EPCAM, collectively indicating that cold exposure potentiates proliferative signaling within the intestinal epithelium (Figure 1K).

### 3.2. Cold Exposure Ameliorates DSS-Induced Colitis in Mice

**Figure 2:**
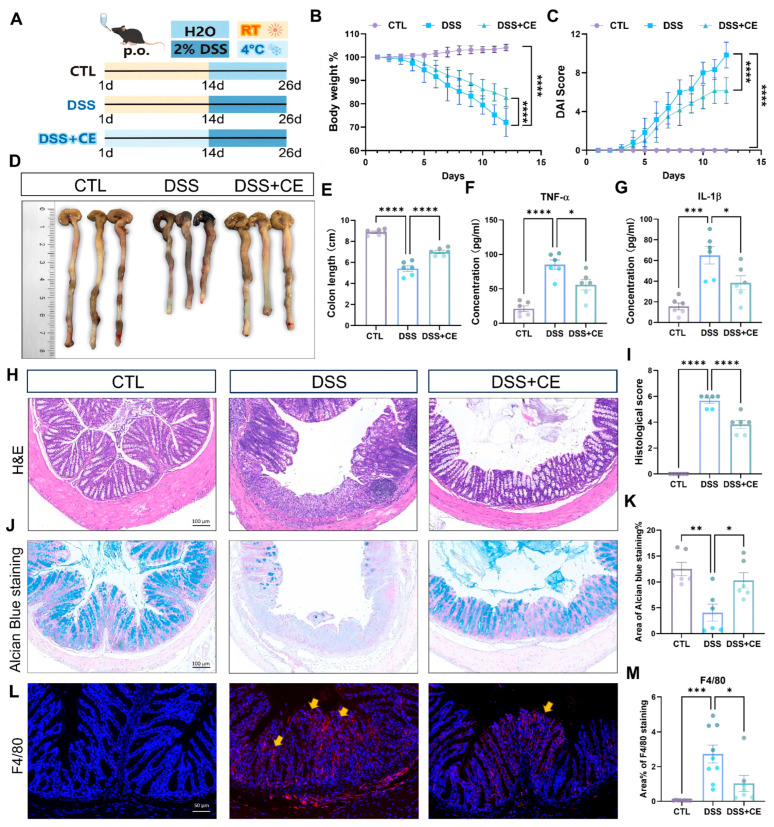
Cold exposure alleviates DSS-induced colitis in mice. (**A**) Experimental timeline illustrating the cold adaptation protocol, DSS administration, and sample collection schedule. (**B**) Dynamic changes in body weight in control, DSS, and DSS + Cold groups (*n* = 6). (**C**) Disease Activity Index (DAI) scores evaluating clinical manifestations of colitis (*n* = 6). (**D**,**E**) Representative images of colon tissues and statistical analysis of colon length from each group (*n* = 6). (**F**,**G**) Serum concentrations of pro-inflammatory cytokines (**F**) TNF-α and (**G**) IL-1β measured by ELISA (*n* = 6). (**H**,**I**) Representative H&E-stained colon sections and histopathological scoring of colon injury (*n* = 5). (**J**,**K**) Representative Alcian blue-stained colon sections and quantification of Alcian blue-positive areas (*n* = 3 and 2–3 fields/n were quantified). (**L**) Representative immunofluorescence images of colon sections stained with macrophage marker F4/80 (red) and nuclear counterstain DAPI (blue), yellow arrows indicate F4/80-positive staining areas. (**M**) Quantitative analysis of F4/80-positive area in colon tissues (*n* = 3 and 2–3 fields/n were quantified). All data represent means ± SEM. Statistical significance was determined by one-way ANOVA or two-way ANOVA, * *p* < 0.05, ** *p* < 0.01, *** *p* < 0.001, **** *p* < 0.0001.

Based on our previous finding that cold exposure promotes intestinal epithelial proliferation, we investigated its potential therapeutic effect in a DSS-induced colitis model. As outlined in Figure 2A, experimental mice were subjected to cold stimulation, while control mice were kept under normal temperature. This was followed by 2% DSS administration in drinking water to all mice, with tissues collected on day 26. We observed that DSS treatment significantly reduced the relative body weight (Figure 2B) and increased the Disease Activity Index (DAI) score (Figure 2C) in mice compared with the normal group, while cold preconditioning remarkably reversed the DSS-induced body weight loss and elevated the DAI score in colitis mice. Morphometric analysis showed that cold treatment prevented DSS-induced colon shortening (Figure 2D,E). ELISA revealed that cold exposure counteracted the DSS-induced elevation of serum pro-inflammatory cytokines TNF-α and IL-1β (Figure 2F,G). Histological examination of H&E-stained colon sections demonstrated that cold exposure ameliorated characteristic DSS-induced damage, including epithelial erosion, crypt loss, and inflammatory infiltration (Figure 2H,I). Alcian blue staining showed that cold treatment restored goblet cell numbers and mucus production, which were diminished in DSS-only mice (Figure 2J,K). Immunofluorescence staining for F4/80 confirmed that cold exposure markedly reduced macrophage infiltration in the colon (Figure 2L,M).

### 3.3. Cold Exposure Promotes Mucosal Repair in DSS-Induced Colitis via Enhanced Epithelial Proliferation

**Figure 3:**
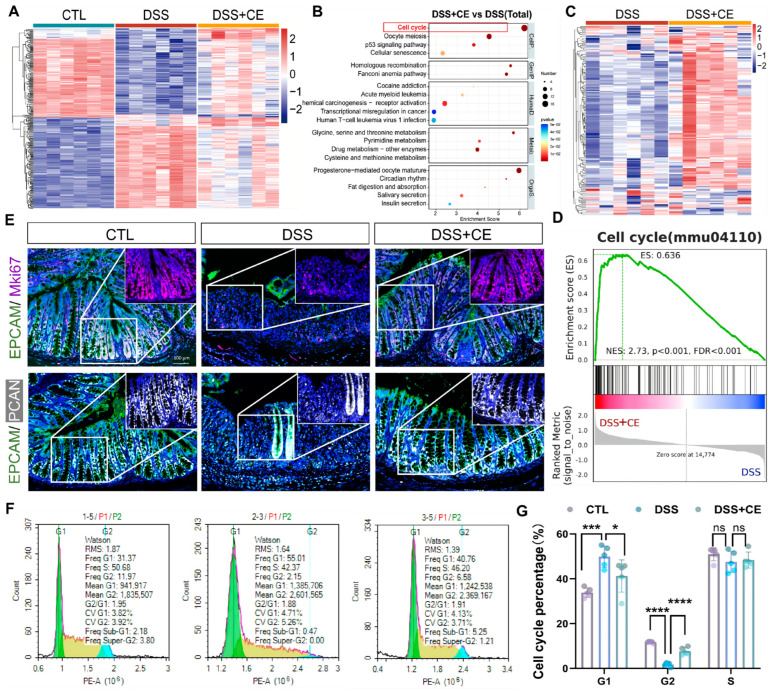
Cold exposure promotes intestinal epithelial regeneration and cell cycle progression in DSS-induced colitis. (**A**) Heatmap of common differentially expressed genes across three-group comparisons. (**B**).Bubble plot of KEGG pathway enrichment analysis in DSS + CE versus DSS comparison. (**C**) Heatmap of cell cycle-related gene expression in DSS versus DSS + CE groups. (**D**) GSEA enrichment plot of the cell cycle pathway in DSS + CE relative to DSS. (**E**) Fluorescence co-staining of proliferation markers Mki67 (pink) and PCNA (white) with intestinal epithelial marker EPCAM (green) in different colonic tissue sections. (**F**,**G**) Cell cycle analysis of isolated colonic epithelial cells. DSS treatment induced G1 phase arrest with significantly increased G1 phase cells and decreased G2 phase cells. Cold treatment significantly reversed these cell cycle alterations (*n* = 5). All data represent the means ± SEM. Statistical significance was determined by one-way ANOVA * *p* < 0.05, *** *p* < 0.001, **** *p* < 0.0001 vs. CTL or DSS group, *ns*, no significance.

As mucosal regeneration represents a key mechanism for colitis resolution, we investigated whether the ameliorative effect of cold exposure (CE) involved enhanced epithelial repair. RNA-Seq analysis of primary colonic epithelial cells revealed distinct transcriptomic profiles among the control (CTL), DSS, and DSS + CE groups (Figure 3A). KEGG pathway analysis identified cell cycle as the most significantly enriched pathway in the DSS + CE versus DSS comparison (Figure 3B). A heatmap of cell cycle-related genes demonstrated systematic upregulation in the DSS + CE group (Figure 3C), The gene list is provided in Supplementary Table S1. Furthermore, this was validated by GSEA showing significant enrichment of the cell cycle pathway (mmu04110; NES = 2.73, *p* < 0.001, FDR < 0.001; Figure 3D).

Immunofluorescence staining confirmed co-localization of proliferation markers Mki67 (pink) and PCNA (white) with epithelial marker EPCAM (green). While DSS treatment markedly reduced signals of all three markers, cold exposure restored their expression (Figure 3E). Cell cycle analysis revealed that DSS treatment induced G1 phase arrest, whereas cold exposure significantly reversed this cell cycle blockade (Figure 3F,G). These results demonstrate that cold exposure counteracts DSS-induced impairment of epithelial proliferation and cell cycle progression, thereby promoting mucosal regeneration in colitis.

### 3.4. Cold Exposure Promotes Intestinal Stem Cell Regeneration

**Figure 4:**
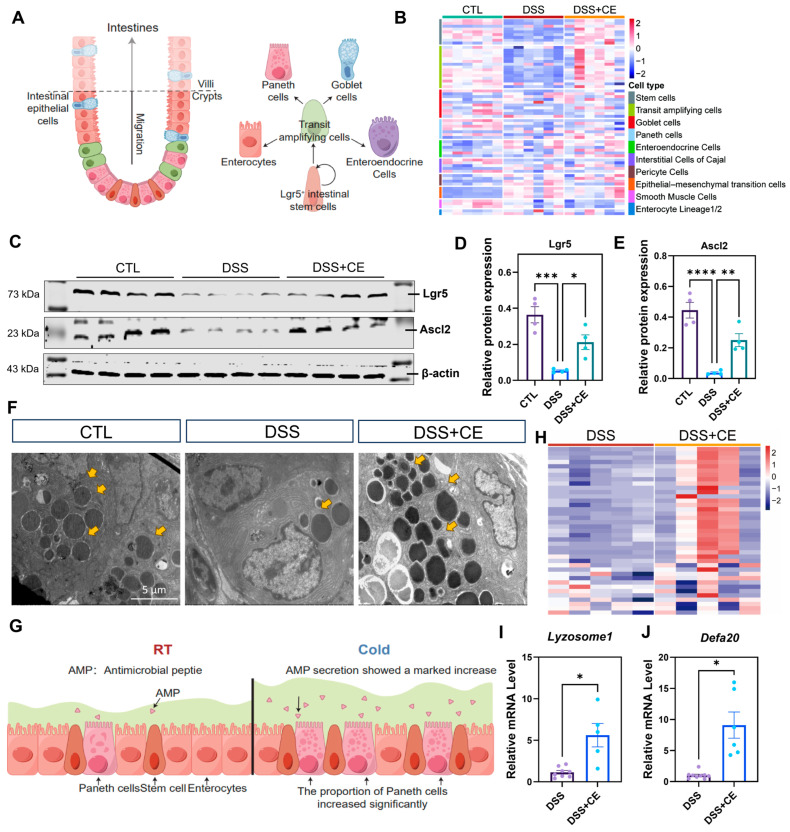
Cold exposure promotes epithelial regeneration and Paneth cell function in DSS-induced colitis. (**A**) Schematic diagram of the differentiation hierarchy in the colonic epithelium. Lgr5-marked stem cells located at the crypt base differentiate into transient amplifying cells, which further give rise to mature epithelial lineages including absorptive enterocytes, goblet cells, Paneth cells, and enteroendocrine cells, collectively maintaining the intestinal physical barrier. (**B**) Heatmap of intestinal epithelial cell marker genes across three experimental groups. (**C**–**E**) Western blot (**C**) and quantification (**D**,**E**) of stem cell markers Lgr5 and Ascl2 in isolated colonic epithelial cells, (*n* = 4). (**F**) Representative TEM images of the secretory granules of Paneth cells. (**G**) Schematic diagram of Cold exposure counteracts DSS-induced barrier damage by improving Paneth cell function and AMP production to reshape microbiota,yellow arrows indicate Paneth cell secretory granules. (**H**) Clustering heatmap of Paneth cell antimicrobial peptide (AMP) genes in DSS versus DSS + CE comparison. (**I**,**J**) qRT-PCR validation of *Lysozyme1* and *Defa20* confirmed cold-induced reversal of DSS-mediated suppression. All data represent the means ± SEM. Statistical significance was determined by one-way ANOVA * *p* < 0.05, ** *p* < 0.01, *** *p* < 0.001, **** *p* < 0.0001.

The aforementioned results demonstrate that cold exposure facilitates mucosal epithelial repair by inducing the proliferation of colonic epithelial cells, which primarily originates from the differentiation of colonic stem cells. As illustrated in Figure 4A, the colonic epithelium is organized into a crypt-based architecture, within which intestinal stem cells reside at the base of the crypts and continuously replenish differentiated epithelial lineages. Located at the base of these crypts are intestinal stem cells (ISCs), which continuously divide to replenish various cell lineages—including absorptive enterocytes, goblet cells, and enteroendocrine cells—constituting a central process in intestinal regeneration. We next analyzed RNA-Seq data to characterize the various intestinal epithelial cell lineages across the three experimental groups (CTL, DSS, and DSS + CE), using previously established cell lineage definitions [23]. These lineages included intestinal stem cells, transit-amplifying (TA) cells, Paneth cells, goblet cells, interstitial cells of Cajal (ICCs), smooth muscle cells (SMCs), and PDGFRα^+^ cells (PCs). As shown in Figure 4B, the expression of marker genes for Lgr5^+^ intestinal stem cells (e.g., *Lgr5* and *Ascl2*) was robust in the CTL group, significantly downregulated in the DSS group, and showed a partial recovery trend in the DSS + CE group. Gene clustering analysis of TA cells revealed that their gene expression was reduced following DSS treatment, and this downregulation was reversed by cold exposure preconditioning. TA cells further differentiate into goblet cells and other lineages. Gene clusters corresponding to these cell types exhibited similar expression patterns: genes significantly downregulated in the DSS group were upregulated again after cold treatment. Together, these transcriptomic findings indicate that DSS treatment markedly disrupts gene expression across intestinal epithelial cell subtypes, while CE intervention partially counteracts the DSS-induced suppression, suggesting that cold exposure promotes the regeneration of multiple epithelial cell populations. The gene list is shown in Supplementary Table S2.

To validate the sequencing data, we isolated colonic epithelial cells and examined the expression of the stem cell marker proteins Lgr5 and Ascl2 by Western blot. The results were consistent with the sequencing analysis: protein levels of Lgr5 and Ascl2 were significantly decreased after DSS treatment, and cold exposure reversed this downregulation, with statistically significant differences (Figure 4C–E).

### 3.5. Cold Exposure Increases Ileal Paneth Cell Secretory Granules and Enhances Antimicrobial Peptide Gene Expression

Intriguingly, our initial phenotypic assessment in Figure 1F revealed that cold exposure led to a significant increase in the total length of the small intestine. This macroscopic adaptation prompted us to further investigate whether systemic cold adaptation also reshapes the innate immune reservoir of the ileal mucosa. As specialized secretory cells, Paneth cells produce antimicrobial peptides (AMPs)—such as lysozyme and defensins—that constitute a key chemical barrier in the gut and are essential for maintaining microbial homeostasis [24,25].

We first examined the ultrastructural changes using transmission electron microscopy (TEM). In normal ileal tissues, Paneth cells exhibited abundant secretory granules with high electron density. DSS administration markedly reduced both the number and staining intensity of these granules. In contrast, cold preconditioning partially restored the granule abundance and electron density within ileal Paneth cells (Figure 4F). Paneth cell dysfunction is commonly observed in intestinal inflammation and is closely associated with an increased risk of IBD [24]. During such inflammatory states, reduced AMP release further weakens innate immune defenses [26]. The morphological improvement induced by cold suggests its potential as a physiological stimulus to reverse this impaired state.

To validate this phenotype at the molecular level, we analyzed the RNA-Seq data from primary mouse small intestinal epithelial cells. Transcriptomic profiling revealed that AMP-related genes—including members of the alpha-defensin family, Reg3 family, and Lysozyme—were significantly upregulated in the cold-exposed group compared to the DSS-only group (Figure 4H; Supplementary Table S3). Notably, alpha-defensins encoded by the *Defa* family serve to reshape the gut microbiota and restrict the colonization of pro-inflammatory taxa [27], while Reg3 family proteins play critical roles in maintaining mucosal integrity [28]. Subsequent qRT-PCR analysis confirmed that cold exposure significantly reversed the DSS-induced suppression of *Lysozyme 1* and *Defa20* expression (Figure 4I,J). Collectively, these results indicate that cold exposure exerts a systemic protective effect by not only promoting colonic regeneration but also enhancing the ileal antimicrobial reservoir, thereby fortifying the integrated intestinal barrier against inflammatory challenge.

### 3.6. Cold Exposure Modulates the Composition of the Gut Microbiota and Promotes the Abundance of Beneficial Bacteria

The aforementioned studies demonstrate that cold stimulation ameliorates intestinal inflammation by regulating the production of intestinal antimicrobial peptides. Given that gut microbiota dysbiosis is a well-established risk factor for intestinal inflammation and a hallmark feature of IBD [26], we further evaluated whether cold exposure reshapes the gut microbial landscape.

**Figure 5:**
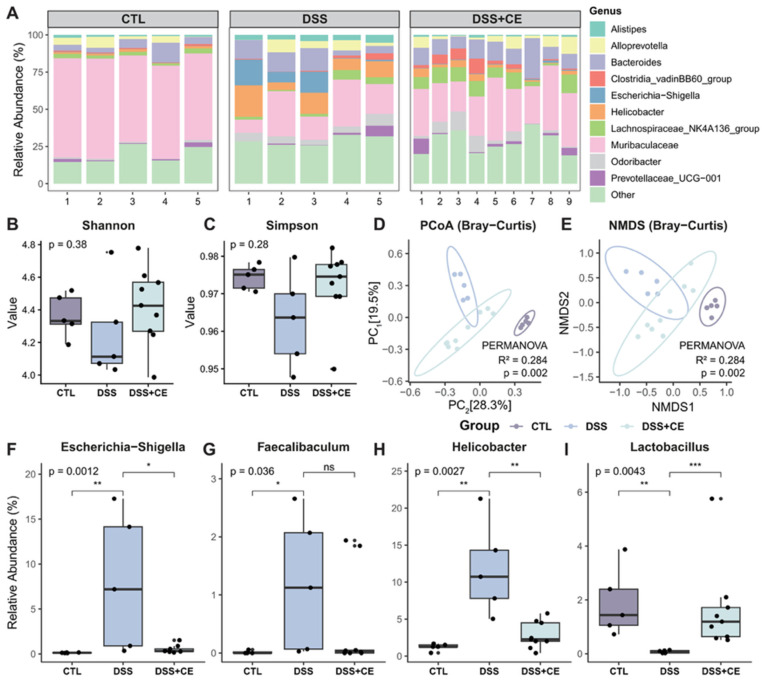
Cold exposure exerts a protective effect by remodeling the gut microbiota and suppressing pro-inflammatory bacteria in DSS-induced colitis. (**A**) Relative taxonomic abundance of the gut microbial community structure. (**B**,**C**) α-Diversity indices including Shannon (**B**) and Simpson (**C**) evaluating microbial richness and evenness among different groups. (**D**–**E**) β-Diversity evaluation using Bray–Curtis distance-based PCoA (**D**) and NMDS (**E**) demonstrated spatial separation and clustering among the CTL, DSS, and DSS + CE groups. (**F**–**I**) Relative abundances of specific inflammation-associated and beneficial genera: *Escherichia-Shigella* (**F**), *Faecalibaculum* (**G**), *Helicobacter* (**H**), and *Lactobacillus* (**I**). DSS treatment markedly enriched opportunistic pathogens and depleted beneficial probiotics. Cold treatment significantly reversed these detrimental microbial alterations (*n* = 5 for CTL and DSS groups; *n* = 9 for DSS + CE group). Statistical significance was determined by the Kruskal–Wallis test followed by the Wilcoxon rank-sum test for pairwise comparisons. *ns*, not significant. *ns*, not significant, * *p* < 0.05, ** *p* < 0.01, *** *p* < 0.001.

We first evaluated the overall microbial community structure. Analysis of the relative taxonomic abundance (Figure 5A) revealed that DSS induction caused profound dysbiosis, characterized by a drastic depletion of *Muribaculaceae*, a dominant symbiotic family in the healthy murine gut. Importantly, cold intervention significantly counteracted this depletion, restoring its abundance. The overall microbial distribution indicated that cold exposure effectively reversed the DSS-induced structural shifts, driving the community profile back toward that of the healthy CTL group. Regarding microbial richness and evenness, α-diversity indices exhibited a decreasing tendency following DSS treatment, which was partially restored in the DSS + CE group (Figure 5B,C). Consistent with these compositional changes, β-diversity analysis using Bray–Curtis distance-based principal coordinate analysis (PCoA) and non-metric multidimensional scaling (NMDS) provided spatial visualization of the microbial communities (Figure 5D,E). Samples within each respective group clustered tightly together, indicating high intra-group consistency and robust reproducibility of our models. Furthermore, a distinct and distant separation was observed between the DSS and CTL groups. Notably, the DSS + CE group clustered intermediately between the CTL and DSS groups. This specific spatial trajectory compellingly visualizes the restorative efficacy of cold exposure on the global microbial architecture disrupted by colitis.

At the genus level, we focused on specific inflammation-associated taxa to further elucidate this protective effect. DSS treatment markedly enriched the relative abundance of several known opportunistic pathogens and pro-inflammatory genera, specifically *Escherichia-Shigella* (Figure 5F), *Faecalibaculum* (Figure 5G), and *Helicobacter* (Figure 5H). Strikingly, cold exposure significantly downregulated the abundance of these harmful bacteria, successfully suppressing their expansion driven by the inflammatory microenvironment. In stark contrast, the beneficial genus *Lactobacillus* (Figure 5I), which plays a pivotal role in maintaining intestinal barrier integrity, was almost entirely depleted in the DSS-treated mice. However, cold preconditioning robustly rescued its abundance, replenishing this crucial probiotic population. Collectively, these findings provide compelling evidence that cold adaptation actively remodels the gut microecology toward a homeostatic state by suppressing colitogenic bacteria and restoring beneficial symbionts, thereby conferring resistance against DSS-induced colitis.

## 4. Discussion

Cold exposure is recognized as an environmental stressor that influences disease pathophysiology by driving metabolic adaptations to maintain energy homeostasis [29]. We previously found that cold alleviates DSS-induced colitis in mice and promotes mucosal repair, though the mechanisms were not fully elucidated [16]. Here, we show for the first time that cold exposure significantly enhances intestinal proliferative signaling in healthy mice, as evidenced by the elevated expression of proliferation markers Mki67 and PCNA in intestinal epithelial cells. Promoting epithelial proliferation is a validated therapeutic strategy for mucosal healing in IBD [30]. While stem cell transplantation can support epithelial regeneration, its clinical use is limited by immune rejection and uncontrolled differentiation risks [31]. Thus, identifying new approaches to activate colonic stem cells and enhance repair remains an important challenge [32].

This study demonstrates that cold exposure promotes colonic epithelial regeneration and accelerates intestinal epithelial cell cycle progression in DSS-induced colitis mice. Sequencing analysis at the cellular subpopulation level revealed upregulated gene expression associated with intestinal stem cells, goblet cells, and Paneth cells in the cold-exposed colitis group compared to the controls. Western blot confirmed the upregulation of stem cell markers Lgr5 and Ascl2. Collectively, these results delineate a previously unrecognized mechanism wherein cold exposure alleviates experimental colitis by activating intestinal proliferative programs and promoting colonic epithelial proliferation to repair the mucosal injury.

**Figure 6:**
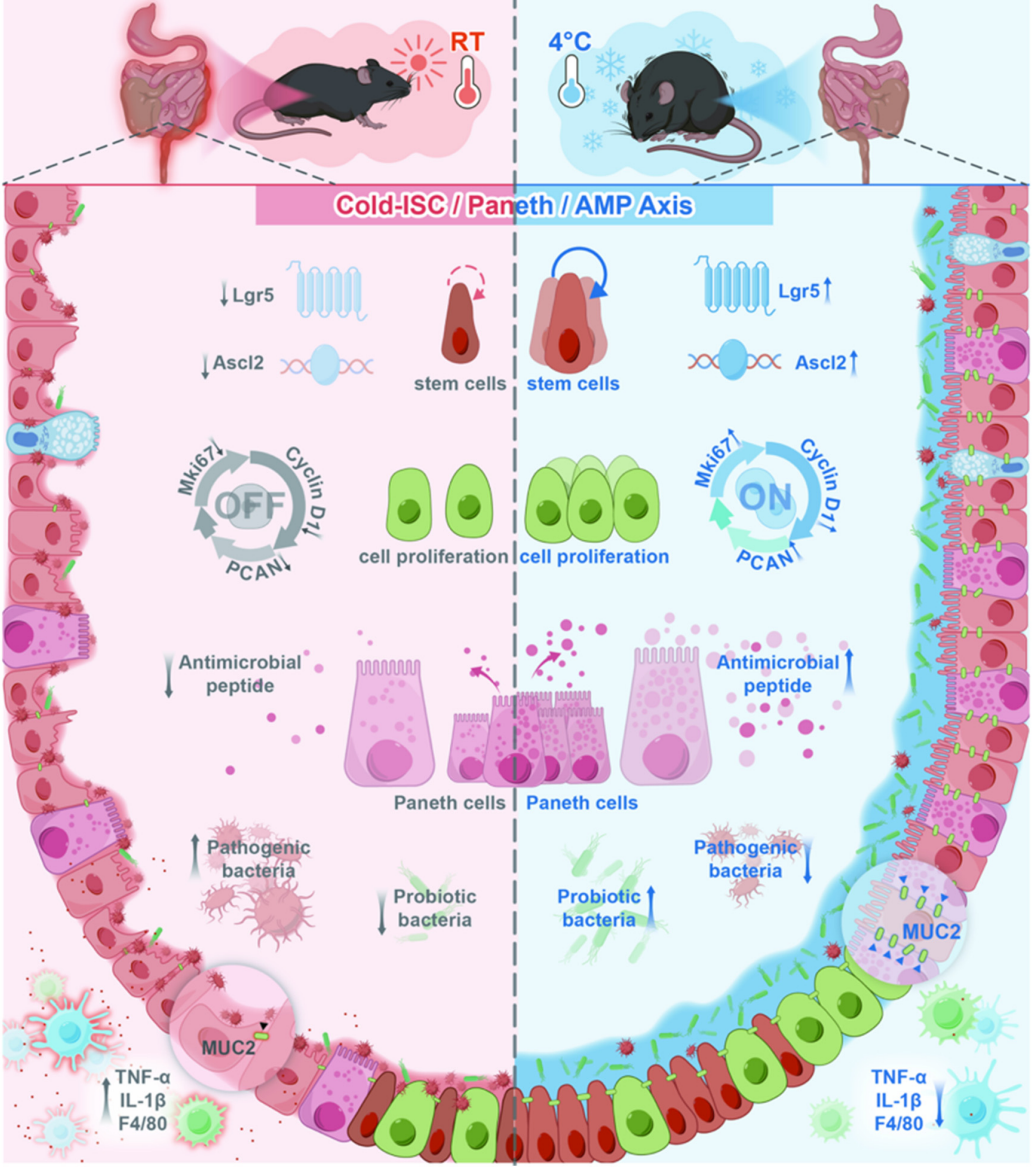
Schematic illustration of how cold exposure alleviates colitis by driving colonic stem cell proliferation and Paneth cell antimicrobial secretion to reshape the gut microbiota.

Furthermore, electron microscopy showed increased secretory granule numbers and higher electron density in Paneth cells from cold-exposed mice. As specialized secretory cells, Paneth cells produce antimicrobial peptides (AMPs)—such as lysozyme and defensins—that constitute a key chemical barrier in the gut and are frequently impaired in inflammatory bowel conditions [33,34]. During intestinal inflammation, Paneth cells are impaired and reduced in number, leading to decreased AMP release. This reduction further compromises the mucus barrier and weakens innate immune defenses [35]. Because Crohn’s disease very frequently affects the terminal ileum, we specifically performed sequencing on ileal epithelial cells. Our results indicated that cold exposure robustly upregulated AMP-related genes, including the *Defa* family, *Reg3* family, and *Lysozyme*. Defa-encoded α-defensins and Reg3 proteins help reshape gut microbiota, reduce pro-inflammatory bacteria colonization, and maintain epithelial homeostasis [27,28,36]. Consequently, by systematically enhancing Paneth cell function in the ileum and inducing a Paneth-like phenotype locally in the colon, cold exposure effectively remodels the disrupted intestinal barrier and reprograms inflammation-associated microbial dysbiosis. These integrated, site-specific mechanisms are schematically summarized in Figure 6.

Recent single-cell transcriptomic landscapes have illustrated that inflammatory stress prompts a definitive phenotypic shift in the colonic epithelium. This remodeling is characterized by the ectopic expression of Paneth cell-specific markers, such as LYZ, within goblet cells localized to the crypt base This molecular shift likely identifies a specialized population known as deep secretory cells (DSCs) [37]. Emerging evidence suggests that DSCs serve as the functional analogues of small intestinal Paneth cells within the colonic niche; by assuming this supportive role, they provide essential signals required to maintain the stem cell microenvironment and coordinate mucosal responses during chronic colitis. Therefore, we characterized the cold-induced acquisition of a “Paneth-like gene expression phenotype” in the colonic epithelium as a localized adaptive response. Rather than initiating a de novo lineage, our data suggest that cold exposure amplifies a compensatory metaplastic program—a mechanism previously documented as a reparative strategy during severe colitis. This regional divergence is further substantiated by our transmission electron microscopy findings: while colonic tissues exhibited localized Paneth-like features, the ileal mucosa showed a marked intensification of secretory granules and AMP production within the native Paneth cell niche. Consequently, we hypothesize that cold exposure exerts parallel, yet site-specific, effects on the intestinal landscape—bolstering endogenous Paneth cell function in the ileum while driving reparative metaplasia in the distal colon to collectively modulate gut microbiota and mucosal integrity. Admittedly, the lack of lineage-tracing evidence (e.g., Lgr5-CreERT2 models) and immuno-electron microscopy remains a limitation in definitively mapping cellular origins. Elucidating the precise developmental trajectories underlying this cold-induced remodeling will be a primary focus of our future mechanistic studies.

Despite these promising findings, our study has several limitations that warrant consideration. First, we exclusively utilized male mice in our experiments. Previous studies have demonstrated that male mice typically exhibit more severe and consistent colitis compared to females [38], which facilitated the establishment of a robust baseline to evaluate the protective effects of cold exposure. However, this approach introduces a potential sex bias [39,40]. Given the known immunomodulatory roles of female sex hormones in gut homeostasis, future studies must incorporate female cohorts to determine whether the cold-induced emergence of colonic Paneth-like phenotypes and ileal Paneth cell activation exhibit sex-dependent disparities. Second, while whole-body cold exposure demonstrates profound therapeutic efficacy in murine models, its direct clinical translation for human IBD patients is currently limited by potential systemic discomfort and cardiovascular stress. Moving forward, future research should focus on deciphering the precise molecular sensors mediating this cold adaptation. Identifying specific thermoreceptors (such as Transient Receptor Potential TRP channels [41]) or downstream metabolic nodes could pave the way for the development of targeted “cold mimetics”—pharmacological agents that replicate the beneficial effects of cold exposure without the need for actual temperature reduction. Exploring these potential pharmacological targets will be crucial for translating our environmental findings into novel, non-invasive adjunctive therapies for IBD.

## 5. Conclusions

Building upon our previous findings, this study demonstrates that systemic cold exposure orchestrates a coordinated protective program along the intestinal axis. Cold adaptation enhances colonic epithelial regeneration by activating proliferative and stemness-associated signaling, while simultaneously reinforcing the ileal antimicrobial reservoir through augmented Paneth cell function and antimicrobial peptide production.

Rather than supporting a definitive lineage conversion, our data indicate that cold exposure promotes a site-specific adaptive remodeling of the intestinal epithelium, integrating regenerative repair in the colon with antimicrobial reinforcement in the ileum.

These findings establish environmental cold adaptation as a non-invasive physiological modulator of the integrated intestinal barrier and provide a conceptual framework linking climatic factors to mucosal regeneration and microbiota homeostasis in inflammatory bowel disease.

## Data Availability

The original contributions presented in this study are included in the article/Supplementary Material. Further inquiries can be directed to the corresponding authors.

## Supplementary Materials

The following supporting information can be downloaded at: https://www.mdpi.com/article/10.3390/biomedicines14030609/s1, Table S1: List of clustered genes related to the cell cycle shown in Figure 3B; Table S2: Gene markers of specific intestinal cell subtypes shown in Figure 4B; Table S3: List of clustered genes shown in Figure 4H; Table S4: Sequences of quantitative real-time PCR (qPCR) primers used in this study.
